# Minimum Dietary Diversity Score and Associated Factors among Pregnant Women at Alamata General Hospital, Raya Azebo Zone, Tigray Region, Ethiopia

**DOI:** 10.1155/2019/8314359

**Published:** 2019-05-02

**Authors:** Kemal Jemal, Mukemil Awol

**Affiliations:** College of Health Sciences, School of Nursing and Midwifery, Salale University, Fitche, Ethiopia

## Abstract

**Background:**

Consumption of diversified food during pregnancy found very important and critical to determine healthy pregnancy outcome. Low dietary diversity has a major adverse effect on mothers, fetus, and life of new born. Dietary diversity is still low in low-resourced countries. Therefore, this study aims to determine prevalence of minimum dietary diversity score (MDDS) and associated factors among pregnant women.

**Methods:**

Facility-based cross-sectional study was conducted from April to May, 2017, in Alamata General Hospital (AGH). Data were collected using a pretested and structured self-interview questionnaire. A systematic sampling technique was used to select study participants. Binary logistic regression and odds ratio with 95% confidence interval (CI) were carried out to see the association between variables and the outcomes.

**Results:**

From a survey of 412 participants, 61.2% had high MDDS and 38.8% had low MDDS. Multivariate analysis revealed that being government employees (AOR = 4.87, CI: 1.70–13.95), merchant (AOR = 4.67, CI: 1.81–12.05), secured food (AOR = 3.85, CI: 2.12–6.97), and eating three meals and above (AOR = 2.66, CI: 1.47–4.82) were significantly associated with high MDDS among pregnant women.

**Conclusions:**

In our study, minimum dietary diversity shows small increment from previous study. Screening and special emphases should be given by a health-care provider on women diet during antenatal follow-up at health-care settings.

## 1. Background

An inadequate dietary diversity is the main concern for pregnant women all over the world. Dietary diversity defined as the number of different food groups consumed over a given reference period [1]. It is the consumption of different food groups within 24 hours prior to assessment [2]. Daily calorie consumption among pregnant women is supposed to be from 2,200 to 2,900 calories [3]. The need for iron is greatly important as it involves the formation of red blood cells and fetal stores [4]. Nonpregnant and pregnant women are requiring iron up to 18 mg/d and 27 mg/d, respectively [5]. During pregnancy, women were required 770 *μ*g/d of vitamin A and 600 *μ*g/d iron foliate which obtained from enriched cereal, dark leafy vegetables, and bread products [6].

In the United States of America, 9.5% of women were inadequate in iron and <1% of the American population were deficient in both vitamin E and foliate [7]. In Pakistan, 86% of pregnant women have medium, 5% have low, and 9% have a high dietary diversity score [8].

In sub-Saharan African countries, diets are predominantly based on starchy foods with little or no animal products and few fresh fruits and vegetables [9]. In Tanzania, cereals and starchy roots contribute 51% and 19% of the total dietary energy supply and 71% and 61% of population use production of maize and rice, respectively [10, 11]. Study in Ghana found that 46.1% pregnant women get their dietary need while 43.9% were not [12]. In Kenya, the prevalence of MDDS was found that 74.5% participants had medium dietary score, 20% of respondents had high dietary score, and 6% had low dietary diversity score [13]. A survey done in Axum, Ethiopia, found that 56.4% and 43.6% had the low and high dietary diversity scores, respectively [14].

A pregnant mother who may not take an adequate nutrient at first trimesters of pregnancy may have high rate of fetal morbidity and mortality [15]. Such insufficient dietary intake may lead to low birth weight, still birth, premature rapture of membrane, intrauterine growth restriction, intrauterine fetal death, and congenital anomalies and affect later on sudden infant death syndrome, developmental impairment, malnutrition, and risk for chronic disease [16–18].

Women who live in developing countries were at high risk for micronutrient akin to vitamins A and D, iron, iodine, folic acid, and vitamins B1, B6, and B12, which may affect the outcome of pregnancy [19–21]. Inadequate consumption of vitamin A during pregnancy can lead to premature birth, eye abnormalities, and impaired vision [22–24].

There were a few studies done in Ethiopia, and little is known on the effect of dietary diversity among reproductive women. To date, there are no studies on the maternal dietary diversity score among pregnant women in the study area. Therefore, the objectives of this study are to [1] determine the prevalence of the maternal dietary diversity score in pregnant women and [2] identify factors associated with pregnant women at AGH.

## 2. Methods

### 2.1. Study Design and Period

An institutional cross-sectional study was conducted from April to May, 2017, in an urban district maternal child health (MCH) clinic.

### 2.2. Study Setting

Alamata is one of the district towns in Rayya Azebo Zone, Tigray region, Ethiopia. It is located in north 600 kilometer from the capital city of Ethiopia (Addis Ababa) and 180 kilometer from the capital city of Tigray region (Mekelle). In Alamata town, there is one general hospital, one health center, and four private clinics. The AGH services a population of greater than 59,915 people with different four major departments including medical, surgical, pediatrics, and obstetrics and gynecology. It also provides outpatient service, emergency, ophthalmology, ART clinic, and MCH clinic.

### 2.3. Study Population

The study population consisted of all pregnant women who were on follow-up at AGH from 16 weeks gestational age, which were included in the sample.

### 2.4. Exclusion Criteria

Pregnant women who were unable to speak/hear and had been seriously ill during data collection were excluded from the study.

### 2.5. Sampling Procedure

The sample size was determined based on a single population proportion formula using Epi Info version 7 with a 95% CI and 5% margin of error and taking 53% proportion of reproductive age women with MDDS that was done in Gojam, Ethiopia [25]. A 10% nonresponse rate was assumed, and 420 sample sizes were estimated. The study participants were selected using a systematic sampling technique. The sampling interval was determined by calculating monthly average attendance for antenatal care follow-up divided by the required sample size, and then, the first study participant was selected randomly.

### 2.6. Data Collection

Pretested and structured self-administered interview questionnaires were used to collect data by trained midwifery professionals. The questionnaire contained three parts. The first part includes sociodemographic characteristics (age, marital status, residence, education, occupation, and others). The second parts were dietary information questionnaires. It contained ten different food groups based on their nutrients: those include grains (white roots, tubers, and plantains), pulses (beans, peas, and lentils), nuts and seeds, dairy, meat (poultry and fish), eggs, dark green leafy vegetables, vitamin A-rich fruits and vegetables, vegetables, and fruits. The third components were food security questionnaires which were categorized into three based on the number of questionnaires they answered (secured food, unsecured food without hunger, and unsecured food with hunger). Women who eat five or more and less than five different food groups have a high and low dietary diversity score, respectively [26, 27].

### 2.7. Data Processing and Analyses

Completed questionnaires were checked for accuracy and completeness in recoding of responses. The data were edited, coded, and cleaned prior to data entry. Data were entered into Epidata version 3.1 and analyzed by using SPSS version 20. Description of means, frequencies, proportions, and rates of the given data for each variable was calculated. Frequencies and percentages for discrete data (noncontinuous) were done. Logistic regression was completed to test for relationship between factors and the dietary diversity of the respondents. A *P* value of <0.05 was used as the criteria for statistical significance.

### 2.8. Ethical Consideration

Ethical clearance was obtained from Woldia University, Faculty of Health Sciences, Department of Midwifery and Research Committee. The permission letter was granted from AGH. Written informed consent was obtained from each study participant. Data collectors were clarified the purpose of the study and assured the confidentiality of the study respondents. To keep privacy of the respondents, the name and any identity were not included in the data collection questionnaire.

## 3. Results

From 420 planned participants, 412 were included in the study with a response rate of 98%. Majority of the participants (34.5%) were unable to read and write while one-fourth of the household were able to write and read, and 28.6% were college and above. Greater than three-fourths of study participants had family size below five and high monthly income (Table 1).

### 3.1. Dietary Intake, Food Security, and Comorbidity Status of the Respondents

A 98.5% of study population had consumed cereals in the period of 24 hours. The main cereal consumed was teff, wheat, and maize that were considered as the staple food within the area. Also, vegetables were an integral part of nutrients containing different minerals and vitamins. Eighty-five percents of study population were consumed dark green leafy vegetables, and 81.6% consumed other vegetables (onion, tomato, and egg plants) (Table 2).

More than two-thirds of the study participants had secured food, and 71.6% of respondents consumed two meals and eating between meals per day. Majority of the respondents (86.9%) had no comorbid disease before 4 weeks of data collection (Table 2).

### 3.2. Factors Associated with High Dietary Diversity

Participants whose education status was college and above were 2.8 times more likely to have high dietary diversity with their diet compared to participants whose education status was elementary school (AOR = 2.8, 95%, CI: 1.90–6.68). Those who had three meals and above had greater odds ratio to have high dietary diversity in their diet compared with those who had two meals and between a meal (AOR = 2.66, 95% CI: 1.47–4.82). Respondents who had secured food were better to have high dietary diversity than those who had unsecured food without hunger (AOR = 3.85, 95% CI: 2.12–6.97). Government employees and merchants have also high dietary diversity in their foods (Table 3).

## 4. Discussion

Our study found that major proportion of pregnant women had insufficient minimum dietary diversity intake: this is a poorly emphasized health problem among pregnant women in Ethiopia. We discovered that three-fifths of pregnant women had high dietary diversity score and 38.8% low dietary diversity score in district urban general hospital during past 24 hours of an interview.

The prevalence of MDDS in this study was higher than the studies conducted in Aksum, Ethiopia; 43.6% had high dietary diversity scores [14]. Also, we found a higher prevalence of MDDS than the studies done in Kenya and Ghana that found the result of 20% and 46% had a high dietary diversity scores consequently [13, 28]. This difference might be due to their study methodology, mainly variation for the food group involved and its food category, while our study contains 10 food groups with two categories, and their study contained nine food groups with three categories. Additionally, geographical location and seasonal variability may be making difference as in Ghana where the study was done at a rural area characterized by poverty and recurrent drought and flood [28].

We found that participants who were college and above level have a good habit of dietary diversity within their diet as compared with those who were elementary school. This might be due to good knowledge about types of food groups that may consume within 24 hours to achieve their dietary diversity level. Women with higher education have a tendency to include a variety of food group and good dietary eating practice in their diet [29]. Less education may be linked with poor food choices and preparation due to lack of knowledge and resources with their dietary diversity [30].

In our study, respondents who had three meals and above had high dietary diversity in their diet compared with those who had two meals and between meals. Conversely, the study done in Pakistan had no association observed between dietary diversity score and nutritional status [8]. This might be related to their economic status. The opportunity of having greater than two meals may have the chance of consuming different categories of meal [31]. Dietary diversity has been strongly associated with socioeconomic status in a household. Individuals with a higher socioeconomic status were found to be consumed a better quality of food diversity than those with a lower socioeconomic status [2, 29].

We also found that secured food have high dietary diversity than those who had unsecured food without hunger. This finding was in line with the result in Malaysia that revealed pregnant women who have secured food were more likely to have a higher dietary diversity score than those who have unsecured food [32]. This might be related to accessibility of adequate food due to better attitude and economic status of respondents. Previous studies show that individuals who had inadequate food and low socioeconomic status may lead to little consumption of diverse diets (fruits, vegetables, and milk products) [33, 34].

As limitation, dietary diversity was assessed based on responses obtained from participants recall, and this depended on memory and their ability to recall accurately. So, recall bias could not be ruled out completely. The 24-hour dietary recall may not truly represent the usual intake.

## 5. Conclusions

This study suggested that around 38.8% of respondents had a low dietary diversity and 61.2% had high dietary diversity mainly defined by inclusion of ten food groups. Regarding determinant factors, being house wife and daily laborer, having household elementary school and unsecured food without hunger, eating two meals and between meals were low dietary diversity. Therefore, measures should be taken on dietary diversity and integrate with primary care in the MCH program.

## Figures and Tables

**Table 1:** Sociodemographic characteristics of study participants in MCH at AGH, Alamata town, 2017 (*n*=412).

Variable	Frequency	Percentage
Age category		
18–29	252	61.2
30–39	139	33.7
40–49	21	5.1
Marital status		
Married	402	97. 6
Widowed	3	0.7
Single	7	1.7
Residence		
Urban	328	79.6
Rural	84	20.4
Family member		
Five and above	86	20.9
Below five	326	79.1
Ethnicity		
Tigray	388	94.2
Amhara	19	4.6
Afar	5	1.2
Age of household		
20–29	79	19.2
30–39	234	56.8
40 and above	99	24.0
Source of drinking water		
Tap water	60	14.6
Pumping water	335	81.3
Protected well	17	4.1
Latrine		
Yes	354	85.9
No	58	14.1
Home gardening practice		
Yes	140	34
No	272	66
Main source of food		
Market	336	81.6
NGO/support	10	2.4
Farmer/garden	66	16
Maternal educational status		
Unable to write and read	143	34.7
Able to write and read	46	11.2
Elementary school	67	16.3
High and preparatory school	92	22.3
College and above	64	15.5
Household educational status		
Unable to write and read	118	28.6
Able to write and read	62	15.1
Elementary school	50	12.2
High and preparatory school	64	15.5
College and above	118	28.6
Occupation		
House wife	183	44.4
Merchant	125	30.4
Government employee	78	18.9
Daily laborer	26	6.3
Monthly income		
0–500	21	5.1
501–1500	66	16.0
1501 and above	325	78.9

**Table 2:** Consumption of food groups, food security, and comorbidity status of study participants in MCH at AGH, Alamata town, 2017 (*n*=412).

Variable	Frequency	Percentage
Starchy staples		
Yes	406	98.5
No	6	1.5
Vitamin A-rich fruit and vegetable		
Yes	168	40.8
No	244	59.2
Other vegetables		
Yes	336	81.6
No	76	18.4
Dark green leaf vegetable		
Yes	348	84.5
No	64	15.5
Fruit		
Yes	92	22.3
No	320	77.7
Meat, poultry, and fish		
Yes	131	31.8
No	281	68.2
Egg		
Yes	141	34.2
No	271	65.8
Pulses		
Yes	213	51.7
No	199	48.3
Dairy		
Yes	203	49.3
No	209	50.7
Nuts and seeds		
Yes	221	53.6
No	191	46.4
Dietary diversity		
High dietary diversity	252	61.2
Low dietary diversity	160	38.8
Food security status		
Food secured	281	68.2
Food unsecured without hunger food	123	29.9
Unsecured with hunger	8	1.9
Food eating pattern		
Three meals and above	102	24.8
Two meals and eating between meal	295	71.6
Two meals only or below	15	3.6
Illness in last 4 week before assessment		
Yes	54	13.1
No	358	86.9
Types of illness		
Malaria	191	46.4
HIV/AIDS	198	48.0
Others	23	5.6

Others: tuberculosis and diabetes mellitus.

**Table 3:** Factors (crude and adjusted odds ratios and confidence intervals) associated with high dietary diversity in MCH at AGH, Alamata town, 2017 (*n*=412).

Variable	Low	High	COR (95% CI)	AOR (95% CI)
Latrine				
Yes	124	230	1	1
No	36	22	0.33 (0.19, 0.59)	0.75 (0.336, 1.677)
Residence				
Urban	116	212	1	1
Rural	44	40	0.50 (0.31, 0.81)	1.30 (0.64, 2.66)
Monthly income				
0–500	9	12	0.70 (0.29, 1.71)	1.60 (0.54, 4.71)
501–1500	39	27	0.36 (0.21, 0.63)	1.06 (0.53, 2.12)
1501 or above	112	213	1	1
Education status				
Unable to write and read	79	64	0.15 (0.07, 0.32)	0.67 (0.24, 2.09)
Write and read	20	26	0.24 (0.99, 0.59)	0.61 (0.19, 1.99)
Elementary school	22	45	0.38 (0.16, 0.88)	1.10 (0.36, 3.33)
High school	29	63	0.40 (0.18, 0.90)	0.68 (0.25, 1.86)
College and above	10	54	1	1
Household education status				
Elementary school	21	29	1	1
Write and read	29	33	0.63 (0.40, 1.74)	1.38 (0.57, 3.30)
Unable to write and read	69	49	0.51 (0.26, 0.98)	1.26 (0.51, 3.11)
High school	20	44	1.56 (0.73, 3.34)	2.31 (0.96, 5.58)
College and above	21	97	3.35 (1.63, 6.88)	2.82 (1.19, 6.68)^*∗*^
Eating pattern				
Two meals and between meal	71	31	1	1
Three meals and above	81	214	0.17 (0.11, 0.28)	2.66 (1.47, 4.82)^*∗*^
Two meals only or below	8	7	0.33 (0.12, 0.94)	3.07 (0.84, 8.21)
Food security				
Food unsecured without hunger	85	38	1	1
Food secured	70	211	6.74 (4.23, 10.75)	3.85 (2.12, 6.97)^*∗*^
Food unsecured with hunger	5	3	1.34 (0.31, 5.91)	1.32 (0.27, 6.59)
Occupation				
Daily laborer	16	10	1	1
House wife	84	99	4.61 (1.66, 12.77)	2.60 (0.80, 8.46)
Merchant	40	85	6.24 (3.25, 9.26)	4.67 (1.81, 12.05)^*∗∗*^
Government employee	14	64	7.65 (6.91, 14.81)	4.87 (1.70, 13.95)^*∗∗*^

## Data Availability

The datasets used and/or analyzed during the current study are available from the corresponding author on reasonable request.

## Abbreviations

AGH: Alamata General Hospital
AIDS: Acquired immune deficiency syndrome
CI: Confidence interval
HIV: Human immune virus
MDDS: Minimum dietary diversity scores
NGO: Nongovernmental Organization.
